# Concomitant progressive supranuclear palsy and Lewy body pathology presenting with circumscribed visual memory loss: A clinicopathological case

**DOI:** 10.1111/bpa.13219

**Published:** 2023-11-05

**Authors:** Christopher Kobylecki, Jennifer C. Thompson, Andrew C. Robinson, Federico Roncaroli, Julie S. Snowden, David M. Mann

**Affiliations:** ^1^ Department of Neurology, Manchester Centre for Clinical Neurosciences Northern Care Alliance NHS Foundation Trust Salford UK; ^2^ Division of Neuroscience, Geoffrey Jefferson Brain Research Centre, Faculty of Biology, Medicine and Health University of Manchester Manchester UK; ^3^ Cerebral Function Unit, Manchester Centre for Clinical Neurosciences Northern Care Alliance NHS Foundation Trust Salford UK; ^4^ Neuropathology Unit, Manchester Centre for Clinical Neurosciences Northern Care Alliance NHS Foundation Trust Salford UK

**Keywords:** dementia with Lewy bodies, neuropsychology, neuropathology, progressive supranuclear palsy

## Abstract

A 70‐year‐old man presented to the clinic with impairment of visual memory and marked predominantly right sided mesial temporal lobe atrophy on imaging. He died 6 years following symptom onset and neuropathological examination showed concomitant progressive supranuclear palsy and Lewy body pathology. Although he did not fulfil clinical criteria for either condition at presentation, we propose that interactions between the two pathologies in mesial temporal regions could result in this atypical clinical phenotype.

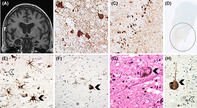

## 1 CLINICAL CASE

A 70‐year‐old right‐handed male presented with a 2‐year history of short‐term memory problems. He was repetitive in conversation, lost track easily and frequently mislaid items, although autobiographical and distant memories appeared preserved. There was no history of hallucinations, apathy, disinhibition or stereotyped behaviour, nor of physical slowing, falls or sphincteric disturbance. He slept for short periods during the day and had episodes of fluctuating arousal. He had a previous history of myocardial infarction, and coronary artery bypass surgery 18 years previously. Cranial nerve examination including eye movements was normal. There were no pyramidal or Parkinsonian signs in the limbs, and gait was normal.

**FIGURE 1:**
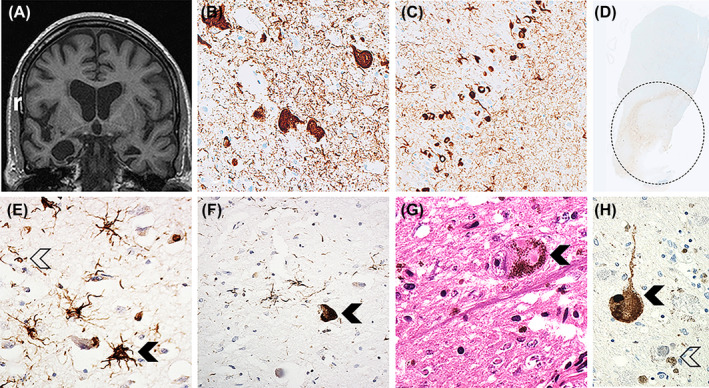
Coronal T1‐weighted MR brain showing marked temporal atrophy, right more than left (A). The temporal cortex shows tau‐positive globoid plaques and a high density of threads (B, AT8 immunoperoxidase—×20); the dentate gyrus showing tau‐positive inclusions with AT8 (C, AT8 immunoperoxidase—×10); Tau‐related pathology affects the pontine tegmentum (circled) (D, AT8 immunoperoxidase—whole mount). Tufted astrocytes (black arrow) and coiled bodies (white arrow) contain 4R Tau (E, immunoperoxidase—×20); 4R‐tau are also present in the dentate nucleus (F, immunoperoxidase—×20). Pigmented neurons in the substantia nigra show Lewy bodies (black arrow) (G, haematoxylin–eosin—×40). Residual neurons in the substantia nigra contain misfolded alpha‐synuclein (black arrow); several melanophages are seen (white arrow) (H, immunoperoxidase—×40).

Neuropsychological evaluation revealed him to be affable, cooperative and socially appropriate. Rate of performance was unremarkable, although occasional lapses in concentration were noted. Mini‐mental state examination (MMSE) score was 30/30. His speech was fluent and grammatically correct. Naming and comprehension were preserved. Perceptuospatial functions, praxis and visual construction were within normal limits. Verbal memory performance was within normal limits, but on visual memory testing he demonstrated marked loss over a delay. Consistent with this, Camden Recognition Memory Test performance was normal for words (24/25, 50th percentile) but chance level for faces (12/25, 5th percentile). Executive function, assessed by the Weigl Colour‐Form Sorting Test, and a locally developed picture sequencing task, was preserved, although maintenance of the Luria 3‐step test was slow and laboured. MRI brain showed marked mesial temporal lobe atrophy, worse on the right compared to left (Figure 1A). His initial clinical diagnosis was Alzheimer's disease (AD), presenting as circumscribed visual memory impairment.

On follow‐up 14 months later, his memory had deteriorated, and he had commenced donepezil 10 mg daily. He would fall asleep intermittently during the day and had occasional episodes suggestive of REM sleep behaviour disorder. His eye movements remained normal and there were no Parkinsonian features on examination. On neuropsychological assessment, there were mild fluctuations in alertness, from which he could be easily roused, and his performance was mildly slowed. Twenty months from original assessment there was mild bilateral limb rigidity, treated with levodopa; his MMSE score was 29/30 and visuospatial functions remained normal. After a further year, he had developed postural instability and falls; there was no vertical gaze palsy. His level of alertness fluctuated during the consultation. MMSE score was 19/30 and there was progressive visuospatial impairment. He died aged 75 years, 6 years following the onset and 6 months following last clinical review.

## 2 NEUROPATHOLOGICAL FINDINGS

*Post‐mortem* examination of the brain showed moderate atrophy, more severe in the hippocampus and amygdala, with preserved grey‐white matter differentiation. The substantia nigra showed bilateral, widespread loss of pigmentation.

Microscopic assessment revealed reactive gliosis and neuronal loss in the neocortical regions, allocortex and amygdala. The substantia nigra showed considerable loss of pigmented neurons, Lewy bodies in some of the spared neurons (Figure 1G), pigment incontinence and melanophages (Figure 1H). The locus coeruleus also showed neuronal loss and free‐lying pigment. Small vessel disease was moderate in severity in the white matter of the centrum semiovale, and mild to moderate in the basal ganglia.

The immunoreaction for phosphorylated tau highlighted mild tau‐related pathology in the anterior superior and medial frontal gyrus, and primary visual cortex whilst the superior and medial temporal gyrus (Figure 1B), and superior parietal gyrus showed moderate density of tufted astrocytes, globoid and flamed neurofibrillary tangles, coiled bodies and deposits in neuritic plaques. Severe tau‐related pathology affected the amygdaloid nuclear complex, the end‐folium (Figure 1C), and CA3–CA1 subfields of hippocampus, and trans‐entorhinal cortex, whilst the subiculum and entorhinal cortex were less affected. Amygdala and uncus were severely affected. In the cerebellum, abnormal tau accumulation was limited to the dentate nucleus. Widespread neuropil threads, globoid tangles, coiled bodies and tufted astrocytes were presented in midbrain and were more prominent in the substantia nigra, red nucleus, oculomotor nucleus, and the reticular formation. Similarly, severe tau‐related pathology was present in the pontine tegmentum examined at the level of the superior cerebellar peduncle including the locus coeruleus (Figure 1D). The medulla examined at the level of the inferior cerebellar peduncle showed tau‐related pathology in the tegmentum, mainly in the reticular formation, and nucleus solitary tract. The olivary nucleus was only mildly involved. Several neurofibrillary tangles, tufted astrocytes and coiled bodies stained for 4R tau (Figure 1E,F) whilst no 3R tau was seen.

Mild to moderate density of β amyloid positive diffuse and neuritic and cored plaque pathology was seen in limbic and parietal cortices but was absent in superior frontal and temporal gyri. The density of neuritic and cored plaques focally exceeds 6 per mm^2^. No deposits were seen in the basal ganglia, brainstem and cerebellum corresponding to CERAD C2 and Thal phase 2.

The immunostain for alpha‐synuclein documented a considerable density of Lewy bodies and neurites in the midbrain including substantia nigra (Figure 1H) and locus coeruleus. The allocortex was affected with a density of Lewy bodies corresponding to Stage 3 according to McKeith criteria [1]. Neocortical Lewy bodies and neurites corresponded to a McKeith stage 2 in the superior and medial temporal gyrus and a grade 1 in the superior frontal gyrus. These features were consistent with high‐risk DLB [2].

The immunostains for TDP‐43 showed normal nuclear expression. No deposition of phosphorylated‐TDP‐43 was observed.

The apolipoprotein E genotype was ε2/ε3.

## 3 DISCUSSION

The initial clinical presentation of circumscribed visual memory disorder in the absence of neurological signs suggested AD rather than DLB or PSP. His initial presentation would not fulfil diagnostic criteria for DLB given the absence of early visuospatial dysfunction or visual hallucinations, which are most strongly predictive for a pathological diagnosis of DLB versus AD [2]. In addition, qualitative neuropsychological features suggestive of a diagnosis of DLB, such as interference and environmental intrusions, were absent at initial assessment but developed as his condition progressed [3]. The subsequent clinical course showed some features that were more consistent with a diagnosis of DLB such as cognitive fluctuations, mild parkinsonism and visuospatial impairment [2]. The relatively late development of falls was the only symptom suggestive of PSP, with eye movement abnormalities absent despite 4R‐tau deposits. He thus did not meet the diagnostic criteria for PSP in life and the cognitive phenotype was dissimilar to that reported in PSP [4].

Pathological features of PSP have been reported in 10 of 60 patients with predominant clinicopathological features of DLB [5]. In addition, Lewy body pathology has been described in cases of PSP [6]. We report a greater extent of neuronal and glial tau pathology than previously documented in DLB. Whereas tau pathology in the entorhinal cortex is reported, the marked neuronal and glial tau pathology in this region was not described in the literature [5]. Other studies suggest that co‐existent DLB and PSP pathology occurs at a frequency similar to age‐matched controls. The detailed clinical and neuropsychological information available in the current report is not often documented in larger pathological series of coexisting DLB and PSP pathology.

Pathological studies have identified atrophy of the amygdala and to a lesser extent hippocampus in both DLB and PSP [7], although marked hippocampal atrophy on MR imaging is more suggestive of AD [2]. The presence of mesial temporal atrophy at presentation is consistent with an initial amnestic presentation, but its extent is atypical given the relatively circumscribed visual memory loss and the underlying pathological substrates. Several studies discussing the coexistence of α‐synuclein and tau pathology have postulated that dual pathologies may interact to influence regional levels of neurodegeneration [5]. Recent work proposed the amygdala, hippocampus and entorhinal cortex as likely anatomical areas for interaction of these two proteins [8]. Similarly, the presence of co‐existent TDP‐43 pathology in cases of PSP has been associated with a higher mesial temporal tau burden and risk of dementia [9]. It is possible to speculate that the combination of tau and Lewy body pathology in limbic regions lead in this case to disproportionate mesial temporal lobe atrophy compared to that normally seen in either DLB or PSP, contributing to an atypical amnestic phenotype.

## AUTHOR CONTRIBUTIONS

**Christopher Kobylecki**: Conception; data acquisition and analysis; Writing of first draft; review and critique; final approval. **Jennifer C. Thompson**: Data acquisition and analysis; writing of first draft; review and critique; final approval. **Andrew C. Robinson**: Data acquisition and analysis; review and critique; final approval. **Federico Roncaroli**: Data acquisition and analysis; review and critique; final approval. **Julie S. Snowden**: Data acquisition and analysis; review and critique; final approval. **David M. Mann**: Conception; data acquisition and analysis; review and critique; final approval.

## CONFLICT OF INTEREST STATEMENT

The authors declare no conflicts of interest.

## ETHICS STATEMENT

Ethical approval for brain donation and access to clinical information was obtained from the Newcastle and Tyneside ethics committee ‘Manchester Brain Bank’ rec ref. 09/H0906/52+5 and ‘Clinical data in research into degenerative brain disease’ rec ref. 09/H0906/53+5. Written informed consent was obtained for both studies.

## Data Availability

Data sharing is not applicable to this article as no new data were created or analyzed in this study.

## References

[1] McKeith IG , Dickson DW , Lowe J , Emre M , O'Brien JT , Feldman H , et al. Diagnosis and management of dementia with Lewy bodies: third report of the DLB Consortium. Neurology. 2005;65(12):1863–1872. 16237129 10.1212/01.wnl.0000187889.17253.b1

[2] McKeith IG , Boeve BF , Dickson DW , Halliday G , Taylor JP , Weintraub D , et al. Diagnosis and management of dementia with Lewy bodies: fourth consensus report of the DLB Consortium. Neurology. 2017;89(1):88–100.28592453

[3] Doubleday EK , Snowden JS , Varma AR , Neary D . Qualitative performance characteristics differentiate dementia with Lewy bodies and Alzheimer's disease. J Neurol Neurosurg Psychiatry. 2002;72(5):602–607. 11971046 10.1136/jnnp.72.5.602 PMC1737879

[4] Hoglinger GU , Respondek G , Stamelou M , Kurz C , Josephs KA , Lang AE , et al. Clinical diagnosis of progressive supranuclear palsy: the movement disorder society criteria. Mov Disord. 2017;32(6):853–864. 28467028 10.1002/mds.26987 PMC5516529

[5] Hishikawa N , Hashizume Y , Yoshida M , Niwa J , Tanaka F , Sobue G . Tuft‐shaped astrocytes in Lewy body disease. Acta Neuropathol. 2005;109(4):373–380. 15668789 10.1007/s00401-004-0967-3

[6] Uchikado H , DelleDonne A , Ahmed Z , Dickson DW . Lewy bodies in progressive supranuclear palsy represent an independent disease process. J Neuropathol Exp Neurol. 2006;65(4):387–395. 16691119 10.1097/01.jnen.0000218449.17073.43

[7] Cordato NJ , Halliday GM , Harding AJ , Hely MA , Morris JG . Regional brain atrophy in progressive supranuclear palsy and Lewy body disease. Ann Neurol. 2000;47(6):718–728. 10852537

[8] Visanji NP , Lang AE , Kovacs GG . Beyond the synucleinopathies: alpha synuclein as a driving force in neurodegenerative comorbidities. Transl Neurodegener. 2019;8:28. 31508228 10.1186/s40035-019-0172-x PMC6727368

[9] Yokota O , Davidson Y , Bigio EH , Ishizu H , Terada S , Arai T , et al. Phosphorylated TDP‐43 pathology and hippocampal sclerosis in progressive supranuclear palsy. Acta Neuropathol. 2010;120(1):55–66. 20512649 10.1007/s00401-010-0702-1 PMC2901929

